# Score de prédiction de récidive après un premier épisode de pneumothorax spontané primitive

**DOI:** 10.11604/pamj.2020.36.107.23432

**Published:** 2020-06-19

**Authors:** Ahmed Ben Saad, Asma Migaou, Maroua Ammar, Saousen Cheikh Mhamed, Nesrine Fahem, Naceur Rouatbi, Samah Joobeur

**Affiliations:** 1 Service de Pneumologie et d’Allergologie, Hôpital Universitaire Fattouma Bourguiba, Rue 1er Juin, 5000 Monastir, Tunisie

**Keywords:** Pneumothorax, récidive, TDM, bulle, Pneumothorax, recurrence, computerized tomography scan, bubble

## Abstract

**Introduction:**

la relation entre les constatations scanographiques et le risque de récidive d’un pneumothorax spontané primitif (PSP) reste conflictuelle. L’objectif de cette étude est de déterminer la relation entre le score scanographique DSS (Dystrophy Severity Score) et la survenue de récidive du PSP après un premier épisode.

**Méthodes:**

il s’agit d’une étude rétrospective incluant les patients hospitalisés pour un premier épisode de PSP entre 2005-2017. Nous avons reparti notre population en 2 groupes, G1: récidive du PSP, G2: absence de récidive. Nous avons procédé à une analyse uni varié incluant différentes variables dont le score DSS suivie d’une analyse multi variée.

**Résultats:**

quatre-vingt-six patients ont été inclus dans cette étude. Quarante-huit pourcent des cas ont eu une récidive du PSP. Bien que le score DSS soit significativement associé à la survenue de récidive du PSP (p=0.008), l'analyse multi variée montre que la présence de bulles à la tomodensitométrie thoracique est le facteur de risque indépendant associé à la récidive du PSP après un premier épisode (rapport des risques: 3.26, p< 0.008).

**Conclusion:**

le risque de récidive d’un PSP est significativement associé à la présence de bulles au scanner thoracique. D’autres études sont nécessaires pour une meilleure appréciation du score DSS.

## Abstract (en)

**Introduction:**

the relationship between computed tomography (CT) scan findings and the risk of recurrence of primary spontaneous pneumothorax (PSP) is controversial. The purpose of this study is to determine the relationship between the Dystrophy Severity Score (DSS) and PSP recurrence after an initial episode.

**Methods:**

we conducted a retrospective study including patients admitted to the hospital with PSP (first episode) between 2005-2017. The study population was divided into 2 groups, G1: PSP recurrence, G2: No recurrence. We undertook univariate analysis including various variables such as the DSS score followed by multivariate analysis.

**Results:**

eighty-six patients were included in this study. Forty-eight percent of cases had PSP recurrence. Although the DSS score was significantly associated with PSP recurrence (p=0.008), multivariate analysis showed that the presence of bubbles on chest CT scan was the independent risk factor associated with PSP recurrence after a first episode (risk report: 3.26, p < 0.008).

**Conclusion:**

the risk of PSP recurrence is significantly associated with the presence of bubbles on chest CT scan. Further studies are needed for better assessment of the DSS score.

## Introduction

Le pneumothorax spontané primitif (PSP) survient essentiellement chez les sujets jeunes [1]. Il met rarement en jeu le pronostic vital, mais le taux de récidive après un traitement conservateur est élevé pouvant atteindre 52% [2, 3]. Il est ainsi associé à une morbidité importante avec un cout non négligeable. La société savante telle que la “British Thoracic Society” (BTS) et l' “American College of Chest Physicians” (ACCP) considèrent la surveillance, l’exsufflation à l’aiguille et le drainage thoracique comme des modalités de traitement conservateurs [4, 5]. Le meilleur traitement qui permet de réduire significativement le risque de récidive homolatérale est la chirurgie. La thoracoscopie ou la chirurgie thoracique vidéo-assisté constituent des alternatives thérapeutiques très prometteuses [6]. Cependant, la stratégie thérapeutique après un premier épisode de PSP est conflictuelle [7]. Il n’existe pas, jusqu’à présent, de stratification universelle du risque de récidive après un premier épisode de PSP [8]. D’autre part, les recommandations actuelles ne situent pas la place de la tomodensitométrie (TDM) thoracique haute résolution (HR) dans ces situations [4, 5]. De ce fait, l’appréciation de ce risque de récidive impliquant une décision thérapeutique constitue un enjeu capital. Ainsi, l’introduction de score scanographique pour apprécier le risque de récidive d’un PSP après un premier épisode et guider les choix thérapeutiques parait nécessaire [9, 10]. Le score DSS ou Dystrophy Severity Score est un outil simple permettant la stratification des patients selon le niveau de risque de récidive [9, 10]. Les études relatives à ce score sont peu nombreuses avec des résultats parfois contradictoires. L’objectif de ce travail est de déterminer la relation entre le score DSS et la survenue de récidive du PSP après un premier épisode.

## Méthodes

**Nature de l’étude:** il s’agit d’une étude rétrospective, mono centrique, analytique portant sur les dossiers des patients hospitalisés pour un premier épisode de pneumothorax spontané (PS) au service de Pneumologie et d’Allergologie à l’Hôpital Universitaire Fattouma Bourguiba de Monastir entre Janvier 2005 et Décembre 2017. Il s’agit d’une étude rétrospective utilisant le registre des patients hospitalisés pour PS et aucune information spécifique permettant d’identifier les patients n’a été utilisée. La confidentialité des données a été maintenue durant toutes les étapes de l’étude.

**Figure 1:**
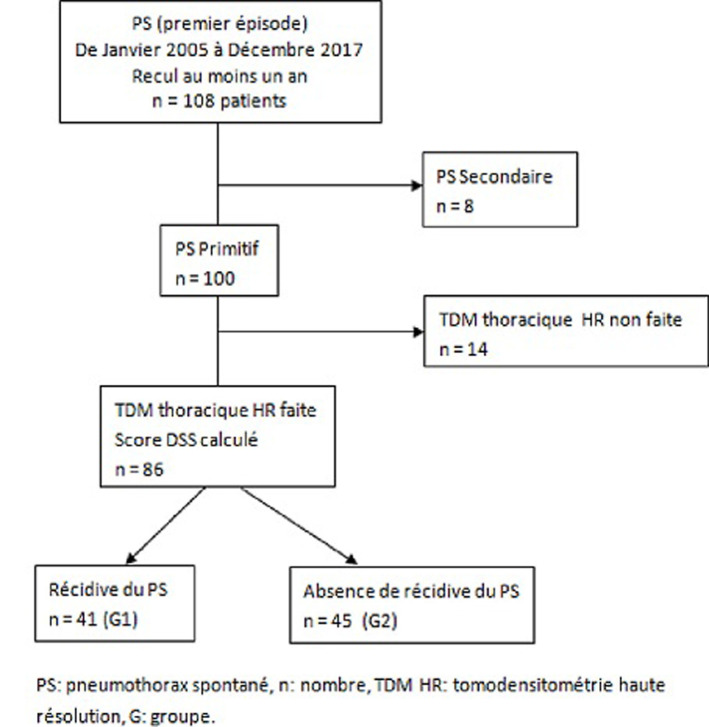
diagramme de flux des patients de l’étude

**Les critères de sélection des dossiers:** les patients inclus dans cette étude sont les malades hospitalisés pour un premier épisode de pneumothorax spontané primitif, ayant bénéficié d’une TDM thoracique HR, avec un suivi d’au moins un an après le premier épisode de pneumothorax. Nous n’avons pas inclus dans cette étude les patients ayant eu un pneumothorax traumatique, iatrogène, ou secondaire à une pathologie pulmonaire sous-jacente (Figure 1).

**Recueil des données:** on a recueilli à partir des dossiers médicaux les données suivants: les caractéristiques démographiques et cliniques de la population: l’âge, le sexe, les antécédents, le tabagisme et la présentation clinique. Les caractéristiques radiologiques: à partir de la radiographie thoracique (le type de pneumothorax, son siège et abondance) et à partir de la TDM thoracique HR (les lésions pulmonaires, leurs sièges, nombre et type). On a retenu les données de deux TDM thoraciques HR: Toshiba 64*2 barrettes et Siemens 16 barrettes. Le scanner thoracique était réalisé dans un délai ne dépassant pas les trente jours suivant le premier épisode. La conduite thérapeutique: repos, exsufflation, drainage thoracique, traitement chirurgical. Les caractéristiques évolutives à court et à long terme.

**Score DSS:** le score DSS est un score scanographique permettant d’évaluer le risque de récidive d’un pneumothorax [10, 11]. Il contient 3 paramètres cotés de 0 à 2. Un seuil de 1cm a été retenu pour différencier entre bulles et blebs. Les bulles avaient un diamètre supérieur à 1cm (Tableau 1). Les patients sont divisés en 3 groupes de risque selon le score: bas risque (0 à3 points), risque intermédiaire (4 à 5 points) et haut risque (6 points).

**Tableau 1:** score DSS

Constatations scanographiques	0 points	1 point	2 points
Type	Absence de lésions	Blebs	Bulle
Nombre	0	Unique	Multiple
Distribution	-	Unilatérale	Bilatérale

**Facteurs prédictifs de récidive:** afin de déterminer les facteurs prédictifs de récidive du PSP après un premier épisode, nous avons défini 2 groupes de patients selon la survenue de récidive ou non durant la période de suivi: groupe 1 (G1): récidive du PS (homo ou controlatérale), groupe 2 (G2): absence de récidive du PS. Nous avons comparé les différents paramètres démographiques, cliniques, radiologiques, thérapeutiques et évolutifs entre les deux groupes.

**Analyse statistique:** les données ont été saisies et analysées grâce au logiciel SPSS (Version 20). Les variables quantitatives sont exprimées en moyennes ± écart type. Les variables qualitatives sont exprimées en taux. La comparaison des variables qualitatives a été faite au moyen du test de Chi2. La comparaison des variables quantitatives a été faite au moyen du test de *Student*. Les facteurs indépendants prédictifs de récidive de pneumothorax ont été déterminés par analyse multi variée (régression Cox). Les facteurs retenus pour l’analyse multi variée étaient tous ceux ayant une association statistiquement significative lors de l’analyse unie variées. Une valeur (p) inférieure à 0.05 était considérée comme statistiquement significative. Les variables ayant une valeur (p) inférieure à 0.2 à l’analyse uni variée ont été incluses dans l’analyse multi variée.

## Résultats

Notre travail a inclus 86 cas parmi 108 patients hospitalisés pour PS durant la période d’étude. L’âge moyen de nos patients était de 39.9 (±18.7) avec une nette prédominance masculine (99%). Le PSP lors du premier épisode était souvent à droite, total et complet. Les bulles ont été retrouvées dans 50% des cas. Soixante-cinq patients ont bénéficié d’un drainage thoracique (Tableau 2). La durée moyenne du suivi après le premier épisode du PSP était de 44.6±28 mois. Quarante-et-un patients (48%) ont présenté une récidive PSP durant la période du suivi. Le délai moyen de récidive était de 15.5 mois. L’analyse unie variée des facteurs prédictifs de récidive du PSP après un premier épisode a inclus des données démographiques, cliniques, radiologiques, thérapeutiques, ainsi que le score DSS. Cette analyse a montré que la présence de bulles à la TDM thoracique et un score DSS élevé (score moyen ou risque intermédiaire et haut) sont significativement associés à un risque de récidive plus élevé (Tableau 3). L’analyse multi variée a montré que la présence de bulles à la TDM thoracique est le facteur de risque indépendant associé à la récidive du PSP après un premier épisode (rapport des risques: 3.26, 95% intervalle de confiance, 1.35-7.85, p < 0.008).

**Tableau 2:** caractéristiques épidémiologiques, cliniques, radiologiques, thérapeutiques et évolutives de la population étudiée

Variable (n = 86)	n (%) ou moyenne (± écart type)
Age (ans)	39.9 (±18.7)
Genre (M)	85 (99%)
Tabagisme	70 (81%)
Intoxication tabagique (PA)	26 (±23)
Siège du PSP (à droite)	60 (70%)
Etendue du PSP (total)	71 (82.6%)
Abondance du PSP (complet)	67 (77.8%)
**TDM thoracique**	
Blebs	7 (8.1%)
Bulles	43 (50%)
Score DSS	2.8 (±2)
Score DSS	
Bas risque (0-3)	41 (48%)
Risque intermédiaire (4-5)	13 (15%)
Risque élevé (6)	32 (37%)
**Traitement**	
Repos et surveillance	9
Exsufflation à l’aiguille	6
Drainage thoracique	65
Pleurodèse chirurgicale	6
Récidive	41 (48%)

n: nombre, M: masculin, PA: paquets-années, PSP: pneumothorax spontané primitif, TDM: tomodensitométrie, DSS: dystrophic severity score

**Tableau 3:** facteurs prédictifs de récidive du PSP après un premier épisode (analyse uni variée)

Variables	G1 (n=41)	G2 (n=45)	p
Age (ans)	40.8	39	0.67
Genre (M,%)	100	98	0.51
Tabagisme (%)	82.9	79.5	0.45
Intoxication tabagique (PA)	31.5	22	0.09
Siège du PSP (à droite,%)	68.3	70.5	0.5
Etendue du PSP (total,%)	80.5	86.4	0.33
Présence de bulles (TDM HR,%)	65.9	36.4	0.006
Score DSS	3.68	2.05	0.008
Score DSS Risque intermédiaire et élevé (DSS > 3) (%)	63.4	34.1	0.006
Drainage thoracique (%)	80.5	70.5	0.2
Durée du drainage (jours)	10.6	12.8	0.23

n: nombre, M: masculin, PA: paquets-années, PSP: pneumothorax spontané primitif, TDM HR: tomodensitométrie haute résolution, DSS: dystrophic severity score

## Discussion

Notre étude avait pour objectif de déterminer la relation entre le score DSS et la survenue de récidive du PSP après un premier épisode. Nous avons considéré différents facteurs prédictifs de récidive du PSP dont le score DSS dans une analyse uni variée puis multi variée. Bien que ce score soit significativement associé à la survenue de récidive du PSP, l’analyse multi variée montre que la présence de bulles à la TDM thoracique est le facteur de risque indépendant associé à la récidive du PSP après un premier épisode. Plusieurs facteurs ont été étudiés dans la littérature pour prédire le risque de récidive d’un PSP. Outre les paramètres cliniques et thérapeutiques, les données de l’imagerie ont fait l’objet de différents travaux avec parfois des résultats divergents. Dans l’étude rétrospective de Casali *et al*. [3] ayant inclus 176 patients, l’analyse multi variée a montré que la présence de blebs ou de bulles au scanner thoracique était significativement associée à la survenue d’une récidive homolatérale (rapport des risques=18). Cependant, un score DSS 3, 4, 5, ou 6 n’était pas associé à un risque de récidive homolatérale statistiquement significative.

Dans notre étude, la présence de bulles au scanner thoracique était aussi le seul facteur indépendant associé à la récidive. Dans un essai randomisé contrôlé multicentrique, les patients avec un premier épisode de PSP ont été classés en deux groupes selon la présence de bulles (≥1cm) ou blebs au scanner thoracique HR. Les patients des deux groupes ont été traité soit par drainage thoracique soit par chirurgie thoracique vidéo-assistée après la randomisation. Pour les patients qui ont bénéficié d’un drainage thoracique, ceux avec des bulles dont la taille était supérieure ou égale à 2cm étaient à plus haut risque de récidive du PSP (rapport des risques=4.4; 95% intervalle de confiance, 1.04-18.83; p=0.03) [9]. Ainsi, les constations scanographiques d’un patient présentant un premier épisode de PSP peut avoir des implications thérapeutiques avec une réduction significative du risque de récidive. D’autres études ont conforté ces constatations. En effet, l’excision des bulles et des blebs avec ou sans pleurodèse a permis de réduire ce risque de récidive de 50% à moins de 15% [3, 11-13]. Cependant, certains auteurs n’ont pas retrouvé de corrélation entre les données de la TDM HR et le risque de récurrence [10, 14, 15]. Cela peut être dû à la taille réduite des échantillons dans ces travaux ainsi qu'une durée limitée de suivi.

Le score DSS a été proposé initialement par Besbes *et al*. [10]. L’étude a inclus 80 patients ayant présenté un premier épisode de PSP avec une durée moyenne de suivi de 34±20 mois. Le taux de récurrence était de 19%. Le score DSS n’était pas significativement associé au risque de récidive. Cependant, d’autres travaux ont conclus à l’intérêt de ce score dans la stratification des patients selon le risque de récidive. L’étude rétrospective de Park *et al*. avait pour objective l’étude du rôle des bulles et des blebs dans la prédiction du risque de récidive homolatérale d’un PSP après un traitement conservateur (repos et surveillance ou drainage) d’un premier épisode de PSP [6]. Ce travail a inclus 299 patients. Le taux de récidive était de 38.2% durant 5 ans. L’analyse par régression de Cox a montré que l’âge et un risque intermédiaire au score DSS étaient les deux facteurs de risque indépendants associés à la récidive. Cependant, un score DSS=6 n’était pas un facteur de risque de récidive indépendant. Primavesi *et al*. ont étudié la relation entre le score DSS et la récidive après un traitement conservateur (groupe B) ou une chirurgie thoracique vidéo-assistée (groupe A) [11]. L’étude a inclus 56 patients avec un taux de récidive de 37.5%. Malgré la taille réduite du groupe B (23 patients), un score DSS intermédiaire ou élevé (DSS 4, 5, ou 6) était associé à un taux élevé de récidive (90%). Un score DSS ≥4 était un facteur de risque indépendant de récidive dans ce groupe (rapport des risques=3.20; 95% intervalle de confiance, 1.11-9.22; p=0.03). Les auteurs soulignent l’intérêt du score DSS dans la prédiction de récidive ainsi que dans l’indication d’un traitement par chirurgie thoracique vidéo-assistée après un premier épisode de PSP.

Néanmoins, certains travaux n’ont pas retrouvé cette association significative entre le score DSS et la récidive du PSP telle que l’étude de Casali *et al*. [3]. Dans un essai randomisé contrôlé, le score DSS a été calculé par rapport au côté du PSP (score DSS homolatéral maximum à 5). Ce score modifié n'était pas associé à un risque de récidive plus élevé contrairement à la taille des bulles [9]. Dans deux autres études prospectives incluant respectivement 35 et 55 patients, le nombre, la taille, et la distribution des bulles n'étaient pas corrélés à la récurrence du PSP [14, 15]. La taille de ces deux échantillons est assez réduite pour pouvoir tirer des conclusions. Ces constatations divergentes pourraient être aussi expliquées par le fait que la rupture de bulles ou de blebs peut se produire indépendamment de la taille ou du nombre des lésions [6].

Notre étude se caractérise par la disponibilité des données ayant permis de calculer ce score malgré qu'elle soit rétrospective. Nous avons un taux de récidive du PSP de 48% durant une période moyenne de suivi satisfaisante. Cela a permis d’avoir deux groupes comparables en termes de nombre. D’autre part, seulement 6 patients ont été traités chirurgicalement après le premier épisode de PSP ce qui n’influence pas non conclusions. Aucun patient n’a bénéficié d’un pleurodèle chimique à travers le drain. Par ailleurs, la TDM thoracique HR a été pratiqué dans 86% des cas de PSP après un premier épisode. Cependant notre travail n’est pas sans limites. D’abord, vu que notre travail est rétrospectif, certains biais de sélections ne peuvent être éliminés. D’autre part, le délai de réalisation du scanner thoracique varie d’un patient à un autre ce qui peut influencer l’appréciation de la taille des lésions pulmonaires en fonction de l’expansion pulmonaire. Nous avons regroupé dans l’analyse uni variée les patients à risque intermédiaire et élevé (DSS >3) vu que le nombre de patients à risque intermédiaire était limité.

Ainsi, le score DSS pourrait jouer un rôle dans la décision thérapeutique des patients se présentant avec un premier épisode de PSP. C’est un score pouvant être fréquemment apprécié en pratique quotidienne avec la large utilisation de la TDM thoracique HR. Cependant, il existe une grande disparité entre les études menées jusqu’à présent en terme de critère d’inclusion, de délai de réalisation du scanner thoracique, de l’approche thérapeutique initiale, et du délai du suivi. Bien qu’il existe des preuves d’efficacité et de sécurité de la chirurgie mini-invasive après un premier épisode de PSP [16, 17], les recommandations actuelles ne suggèrent ni la réalisation systématique d’un scanner thoracique HR ni la large indication de la chirurgie thoracique vidéo-assistée après un premier épisode [4, 5, 18, 19]. D’autre part, d’autre facteurs sont à prendre en considération dans l’interprétation de tels scores. En effet, le risque de récidive homo ou controlatérale pourrait dépendre du coté ou il existe le plus de lésions. La poursuite de l’intoxication tabagique est aussi un facteur déterminant dans la récidive du PSP [20]. Ces hypothèses sont attestées par la récidive du PS après résection chirurgicale de blebs sans pleurodèse associée [21-23]. De ce fait, le risque de récidive ne dépend pas seulement de la rupture de bulles ou de blebs.

## Conclusion

Dans notre étude, bien que le score DSS soit significativement associé à la survenue de récidive du PSP, l’analyse multi variée a montré que la présence de bulles à la TDM thoracique HR est le facteur de risque indépendant associé à la récidive du PSP après un premier épisode. D’autre études prospectives, avec un nombre considérable d’inclusion et un délai de suivi prolongé sont nécessaires pour une meilleure appréciation de ce score, son intérêt dans les décisions thérapeutiques, sa validation, et éventuellement son incorporation dans les recommandations.

### Etat des connaissances sur le sujet

- La relation entre les constatations scanographiques et le risque de récidive d’un pneumothorax spontané primitif (PSP) reste conflictuelle;
- Quelques scores ont été établis pour étudier le risque de survenue de récidive du PSP après un premier épisode.

### Contribution de notre étude à la connaissance

- Bien que le score DSS soit significativement associé à la survenue de récidive du PSP, l’analyse multi variée montre que la présence de bulles à la tomodensitométrie thoracique est le facteur de risque indépendant associé à la récidive du PSP après un premier épisode;
- A notre connaissance, il s’agit du premier travail étudiant ce score dans une population africaine.
